# Effects of Asymmetric Auditory Deprivation in Sequential Cochlear Implant

**DOI:** 10.1002/ohn.70308

**Published:** 2026-06-03

**Authors:** Eduardo S. Farinazzo, Rubens V. de Brito Neto, Elisabete H. Yamaguti, Marco A. Fornazieri, Luiz F. M. Lourençone

**Affiliations:** ^1^ Cochlear Implant Section and Department of Otolaryngology, Hospital for Rehabilitation of Craniofacial Anomalies University of São Paulo Bauru Brazil; ^2^ Department of Otolaryngology University of São Paulo School of Medicine São Paulo Brazil; ^3^ Department of Otolaryngology State University of Londrina Londrina Brazil; ^4^ Departament of Medicine Bauru School of Medicine‐FMBRU/USP Bauru Brazil

**Keywords:** auditory deprivation, sequential cochlear implant, speech recognition

## Abstract

**Objective:**

To evaluate the influence of auditory deprivation on sequential cochlear implants in postlingual patients, comparing the ear with shorter deprivation (ESAD), longer deprivation (ELAD), and the bilateral condition (BIL).

**Study design:**

Retrospective study.

**Setting:**

A tertiary referral center.

**Methods:**

Postlingually deafened patients who underwent sequential bilateral implantation between 1990 and 2021. Auditory performance was assessed over 24 months after activation of each implant. Outcomes included free‐field pure‐tone average (PTA), speech detection threshold (SDT), and sentence recognition in quiet and in noise at +10 dB SNR. Secondary variables were interimplant interval, duration of preimplant hearing aid use, etiology, and electrode insertion depth.

**Results:**

ESAD outperformed ELAD in quiet (73% [IQR 44‐92] vs 37% [IQR 0‐87]) and in noise (27% [IQR 0‐62] vs 0% [IQR 0‐45]) at 24 months (*P* < .001). BIL achieved superior results to both monaural conditions in quiet (97% [IQR 72.5‐100], *P* < .001) and in noise (57% [IQR 23‐86.5], *P* = .008), regardless of ESAD experience. Interimplant interval correlated positively with BIL performance (*r* = 0.450; *P* < .001). Longer hearing aid use was negatively associated with ESAD (*r* = −0.293; *P* = .020) and BIL performance (*r* = −0.312; *P* = .018). Partial electrode insertion in ELAD was linked to poorer BIL outcomes in quiet (*P* = .045). No correlations were found for age or etiology.

**Conclusion:**

Shorter auditory deprivation and interimplant intervals are associated with better outcomes. Bilateral stimulation enhances performance even with long deprivation in 1 ear, and auditory experience with the first implant improves results. Complete insertion favors the more deprived ear, whereas prolonged ineffective hearing aid use may compromise outcomes.

Among the various factors influencing hearing outcomes in cochlear implant (CI) users, the duration of auditory deprivation remains one of the primary sources of variability. Prolonged absence of auditory stimulation can impair speech perception, reduce neural plasticity, and limit the overall benefit derived from the device, particularly in adults with severe‐to‐profound sensorineural hearing loss.

Although technological advancements have significantly enhanced CI performance over recent decades, clinical outcomes still exhibit substantial variability. This heterogeneity is attributed to both device‐specific and patient‐related factors. Among the individual variables, the duration of auditory deprivation, age of onset (prelingual vs postlingual), presence of residual hearing, prior use of amplification, etiology of hearing loss, duration of CI use, and psychosocial support have all been identified as important contributors.^1^, ^2^

In the context of sequential bilateral cochlear implantation, the asymmetry in auditory deprivation between ears offers a unique model for isolating the effects of deprivation itself. Assessing each implanted ear separately, as well as in the bilateral listening condition, provides valuable insight into how asymmetric auditory input impacts speech recognition over time. Previous studies suggest that, despite prolonged deprivation in 1 ear, bilateral stimulation can yield substantial functional gains, particularly when auditory experience with the first implant is greater.^3^, ^4^

The objective of this study is to evaluate the influence of auditory deprivation time on speech recognition outcomes in postlingually deafened individuals with sequential bilateral CIs. Specifically, speech recognition performance in quiet and in noise is compared among the ear with the shortest auditory deprivation (ESAD), the ear with the longest auditory deprivation (ELAD), and the bilateral condition (BIL) over a 24‐month follow‐up period.

## Materials and Methods

### Study Design

This was a longitudinal, retrospective study that analyzed the medical records of individuals with sequential bilateral CIs and nonsimultaneous activation, who underwent regular otorhinolaryngologic and audiologic follow‐up at the Cochlear Implant Section of the Hospital for Rehabilitation of Craniofacial Anomalies, University of São Paulo (HRAC/USP), Bauru campus, between 1990 and 2021. The study was approved by the HRAC/USP Research Ethics Committee (protocol no. 76777223.9.0000.5441).

### Participant Eligibility

All included participants were postlingually deafened. Exclusion criteria comprised auditory neuropathy, cochlear nerve hypoplasia, cochlear malformations, middle ear pathologies such as cholesteatoma, vestibular schwannoma, and revision surgeries due to internal device failure.

Auditory deprivation (duration of deafness) was defined as the duration of severe‐to‐profound sensorineural hearing loss in each ear prior to cochlear implantation, following definitions commonly adopted in the CI literature.^2^ Continuous hearing aid use up to the time of implantation was considered indicative of ongoing auditory stimulation, whereas in cases where hearing aid use was discontinued, auditory deprivation was defined as the interval between cessation of amplification and cochlear implantation. This operational definition was chosen to provide a consistent and reproducible estimate of deprivation across a long retrospective study period.

### Data Collection

The implanted ear with the shortest auditory deprivation and the ear with the longest deprivation were assessed individually and in the bilateral condition at the following time points: preoperatively and at 3, 6, 12, and 24 months after CI activation. At 24 months, the auditory performance of the BIL condition was compared with that of the ESAD, which had the shortest deprivation period and the longest CI experience.

Pure‐tone averages (500‐4000 Hz) and speech detection thresholds (SDT) in the free field were measured preoperatively and at each follow‐up time point for both ESAD and ELAD.

Speech perception was assessed using a sentence recognition test in quiet and in noise.

For the preimplant assessment, speech perception testing was performed in the free field with patients wearing their hearing aids and was used to characterize functional auditory benefit from amplification prior to cochlear implantation. All patients met national CI candidacy criteria established by the Brazilian Ministry of Health, which are conceptually aligned with internationally accepted indications of insufficient functional benefit from hearing aids despite appropriate amplification.^5^

The test consisted of 20 recorded affirmative sentences in Portuguese, each containing 3‐7 words, totaling 100 unique words. The lists were phonemically balanced according to the Brazilian Portuguese phonetic inventory. For the noise condition, a competing noise was presented at 50 dB SPL, yielding a +10 dB signal‐to‐noise ratio (SNR), using the same loudspeaker. Participants were instructed to repeat the stimuli, and each correctly repeated word was scored, with final scores ranging from 0% to 100%.^6^

### Subgroup Analysis

A subgroup analysis stratified ears according to deprivation duration (≤10 vs >10 years). Because deprivation duration is inherently ear‐specific and asymmetric in sequential implantation, analyses were conducted at the ear level. Stratification by implanted side (ESAD vs ELAD) was not feasible due to insufficient sample size in certain subgroups, particularly ESAD ears with deprivation >10 years (n = 4), which would preclude reliable statistical comparison.

### Postoperative Management

Postimplantation auditory rehabilitation was conducted according to standardized clinical protocols at the tertiary referral center throughout the study period. CI activation was routinely performed approximately 30 days after surgery. After activation, patients underwent regular otolaryngologic follow‐up visits combined with CI programming (mapping) conducted by experienced speech‐language pathologists.

Mapping sessions were scheduled every 3 months during the first year after activation, every 6 months during the second year, and annually or biennially thereafter, depending on individual clinical needs and device stability.

All patients were referred for structured postimplant auditory rehabilitation (speech therapy), focusing on auditory training and speech perception development. Although specific therapeutic materials evolved over the study period, the overall rehabilitation framework and follow‐up schedule remained consistent.

### Statistical Analysis

Descriptive statistics for nonnormally distributed data were reported as medians and interquartile ranges (IQR, 25th‐75th percentile). Categorical variables were presented as absolute frequencies and percentages.

For normally distributed quantitative variables (free‐field PTA and SDT), a two‐way repeated‐measures ANOVA was applied, considering evaluation time points and ear (ESAD vs ELAD) as factors. Bonferroni correction was used for multiple comparisons.

Nonnormally distributed variables were analyzed using the Wilcoxon signed‐rank test (ESAD vs ELAD) and Friedman's ANOVA for repeated measures across time points. When appropriate, Dunn's post hoc test was applied.

Spearman correlations and Mann‐Whitney Test were used to explore associations between predictor variables and outcomes in quiet and noise sentences recognition. A subgroup analysis stratified ears by deprivation duration (≤10 vs >10 years), between‐group comparisons at each time point were performed using the Mann‐Whitney *U* test.

Statistical significance was set at *P* ≤ .05. Analyses were performed using SPSS version 22.0 and GraphPad Prism 8.

Missing data were handled using a complete‐case approach for each specific analysis. Patients with unavailable measurements at a given follow‐up time point were excluded from that particular longitudinal comparison but remained included in analyses for which complete data were available. No data imputation procedures were performed.

## Results

**Figure 1:**
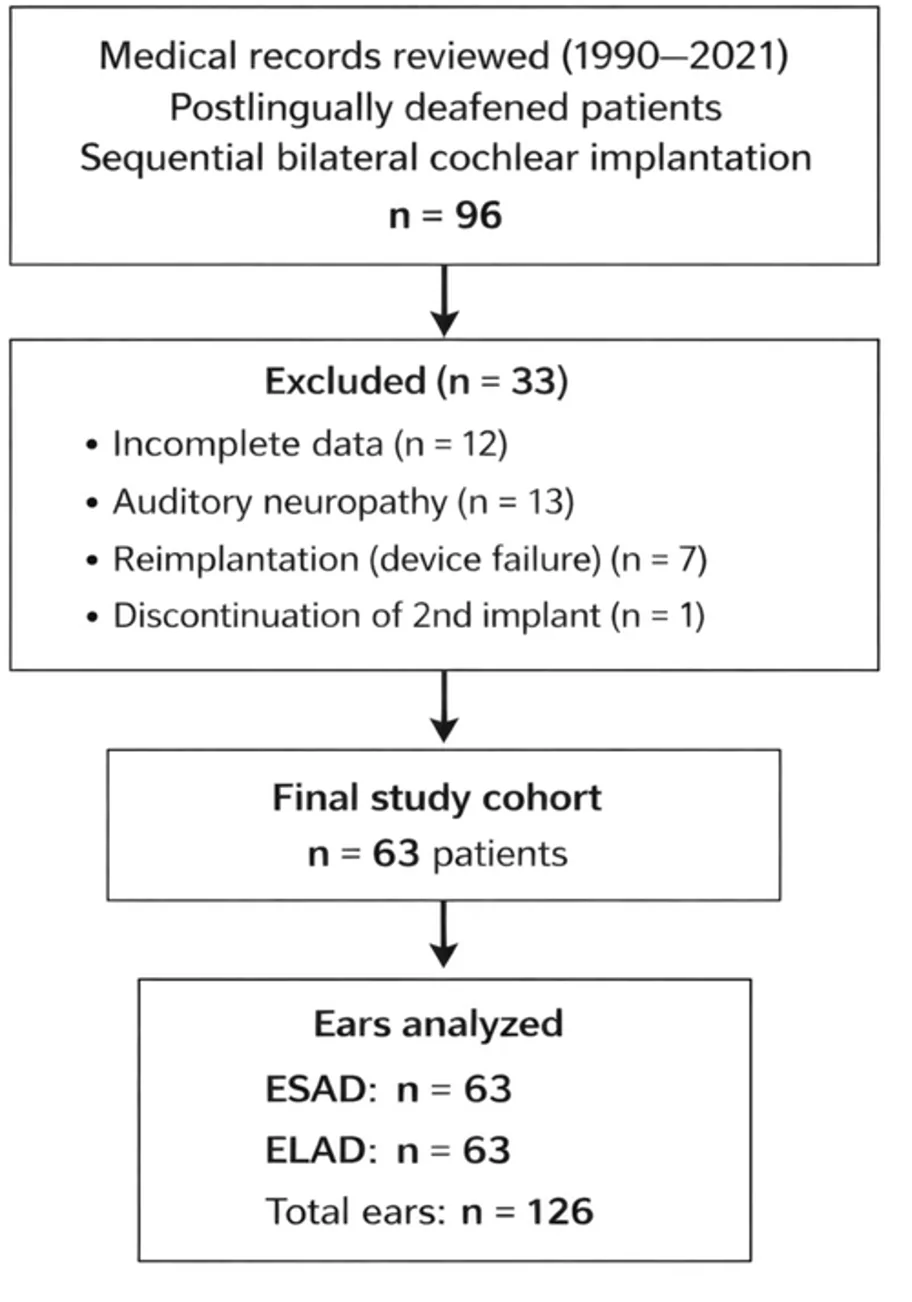
Flow diagram of patient selection. Flow diagram summarizing patient selection for sequential bilateral cochlear implantation (1990‐2021). Of 96 records reviewed, 33 were excluded. The final cohort comprised 63 patients (126 ears). ELAD, ear with longer auditory deprivation; ESAD, ear with shorter auditory deprivation.

A total of 96 medical records of postlingually deafened who underwent sequential bilateral cochlear implantation were initially reviewed. Of these, 33 were excluded: 12 due to incomplete data, 13 due to a diagnosis of auditory neuropathy, 7 due to reimplantation following internal device failure, and 1 due to discontinuation of the second implant caused by pain and local edema. The final study sample comprised 63 patients (Figure 1).

### Population Characterization

As shown in Table 1, the median auditory deprivation time for the ear with the shortest deprivation was 0 months (IQR 0‐24), while for the ear with the longest deprivation was 132 months (IQR 24‐288) (*P* < .001). The median duration of hearing aid use prior to cochlear implantation was 84 months for ESAD (IQR 12‐216) and 24 months for ELAD (IQR 0‐168) (*P* = .433).

**Table 1:** Population Characterization

		ESAD (n = 63)	ELAD (n = 63)	*P*‐value
Auditory deprivation (months)^a^		0 (0‐24)	132 (24‐288)	<.001
Time of previous HA use (months)^a^		84 (12‐216)	24 (0‐168)	.433
Insertion of total electrodes (%)		57 (90.5)	60 (95)	.453
PTA without HA Pre (dB HL)^a^		106 (100‐113)	113(105‐118)	.016
SDT without HA Pre (dB HL)^a^		90 (80‐100)	95 (85‐100)	.004
PTA with HA Pre (dB HL)^a^		70 (61‐84)	74 (66‐95)	.068
SDT with HA Pre (dB HL)^a^		45 (40‐60)	50 (40‐73)	.091
Age (years)^a^		32.83 (13.58‐ 46.33)	42 (23‐52.42)	
Male (%)	27 (42.9)			
ESAD experience time when BIL at 24 months (months)^a^	114(75.5‐168)			
Interimplant time (months)^a^	84(40‐144)			
**Manufacturing Company**				
Advanced bionics (%)	18 (28.6)			
Cochlear (%)	14 (22.2)			
Medel (%)	26 (41.3)			
Oticon (%)	5 (7.9)			
**Internal devices**				
Clarion C1 (Enhanced Bip/12) (%)		1 (1.6)	0	
C40+ (%)		6 (9.5)	0	
CI22M (%)		1 (1.6)	0	
CI24RE (CA) (%)		6 (9.5)	7 (11.1)	
CI24RST(%)		2 (3.2)	0	
CI422 (SRA) (%)		5 (7.9)	6 (9.5)	
CI522 (%)		0	1 (1.6)	
Concerto 2 Form 24 (%)		1 (1.6)	6 (9.5)	
Concerto Medium (%)		3 (4.8)	4 (6.3)	
Concerto Compressed (%)		0	1 (1.6)	
Concerto Flex 28 (%)		0	1 (1.6)	
Digisonic SP EVO (%)		5 (7.9)	0	
Hires 90 K HiFocus 1 J (%)		6 (9.5)	0	
Hires 90 K Adv. 1 J (%)		2 (3.2)	1 (1.6)	
Hires 90 K Adv. HiFocus MS (%)		5 (7.9)	4 (6.3)	
Hires 90 K MS (%)		1 (1.6)	1 (1.6)	
Hires Ultra HiFocus MS (%)		2 (3.2)	6 (9.5)	
Hires Ultra HiFocus SlimJ (%)		1 (1.6)	5 (7.9)	
Hires Ultra 3D HiFocus SlimJ (%)		0	1 (1.6)	
Pulsar CI 100 (%)		1 (1.6)	0	
Sonata compressed (%)		3 (4.8)	7 (11.1)	
Sonata medium (%)		3 (4.8)	4 (6.3)	
Sonata standard (%)		9 (14.3)	3 (4.8)	
Neuro Zti EVO (%)		0	5 (7.9)	

Data presented with N and percentage.

Abbreviations: BIL, bilateral condition; ELAD, ear with longer auditory deprivation; ESAD, ear with shorter auditory deprivation; HA, hearing aids; PTA, pure tone average; SDT, speech detection threshold.

^a^ Median (IQR, 25th‐75th).

The etiology of hearing loss was unknown in 60.3% of cases (n = 38), 19% (n = 12) attributed to meningitis. Other identified etiologies included otosclerosis, trauma, ototoxicity, infections, genetic syndromes, and autoimmune disorders.

Preoperative audiometric thresholds (500‐4000 Hz) were significantly lower in the ESAD ear (median 106 dB HL [IQR 100‐113]) compared to the ELAD ear (median 113 dB HL [IQR 105‐118]) (*P* = .016). The mean aided free‐field thresholds prior to surgery were 70 dB HL (IQR 61‐84) for ESAD and 74 dB HL (IQR 66‐95) for ELAD (*P* = .068).

Pre‐implant sentence recognition in quiet and in noise, assessed with hearing aids, was absent in most patients, with median scores of 0% (IQR 0‐0) for both ESAD and ELAD, indicating minimal functional speech understanding and insufficient benefit from amplification.

### Comparative Hearing Performance

**Figure 2:**
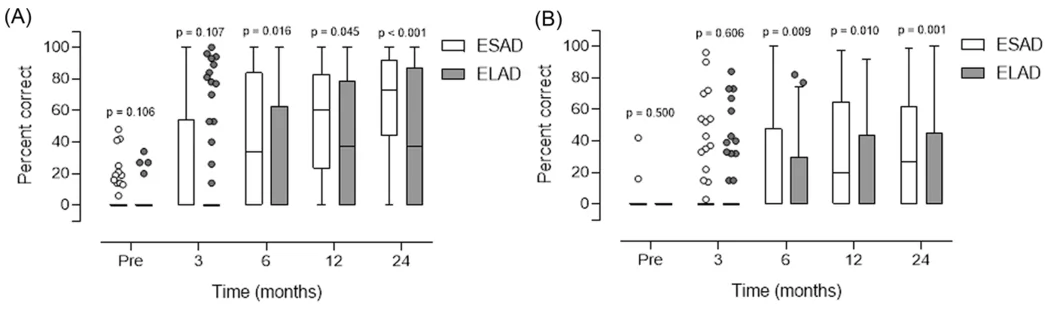
Longitudinal speech recognition performance. Sentence recognition in quiet (A) and noise (B) over 24 months comparing ears with shorter (ESAD) and longer (ELAD) auditory deprivation, demonstrating consistently better outcomes for ESAD.

**Figure 3:**
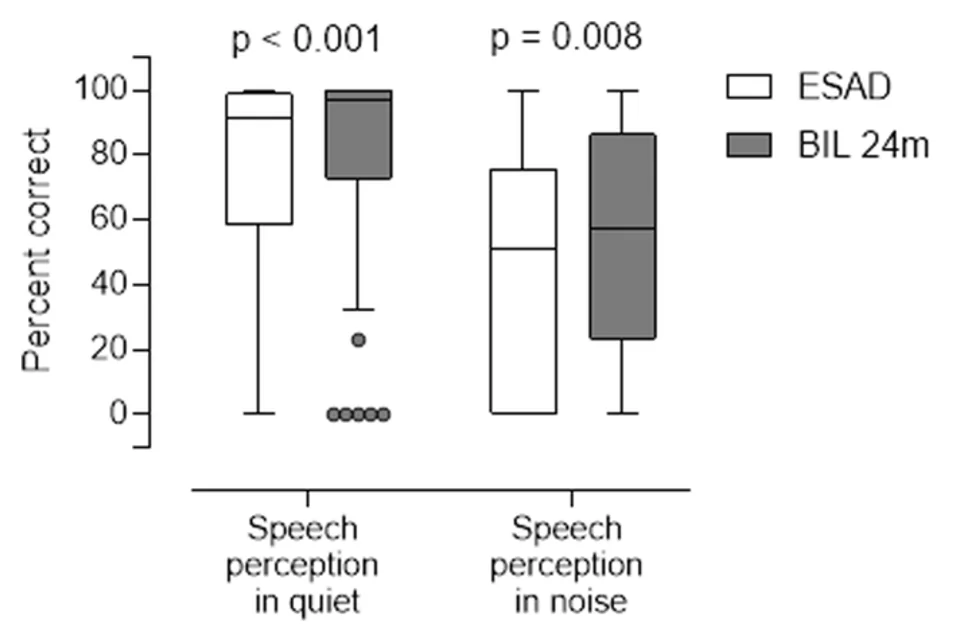
Bilateral superiority at 24 months. Sentence recognition in quiet and noise shows superior performance in the bilateral condition (BIL) compared with the ear with shorter auditory deprivation (ESAD).

ESAD demonstrated significantly better auditory performance than ELAD (Figure 2). Additionally, the combined use of both implants yielded further auditory benefit (Figure 3), regardless of the degree of auditory deprivation or the duration of CI use on the ESAD side (median use time: 114 months [IQR 75.5‐168]).

At the 24‐month follow‐up, the median sentence recognition score in quiet was 91% for ESAD (IQR 58‐99) and 97% for BIL (IQR 72.5‐100) (*P* < .001). In noise, the corresponding medians were 51% for ESAD (IQR 0‐76) and 57% for BIL (IQR 23‐86.5) (*P* = .008).

### Longitudinal Evolution of Auditory Performance

**Figure 4:**
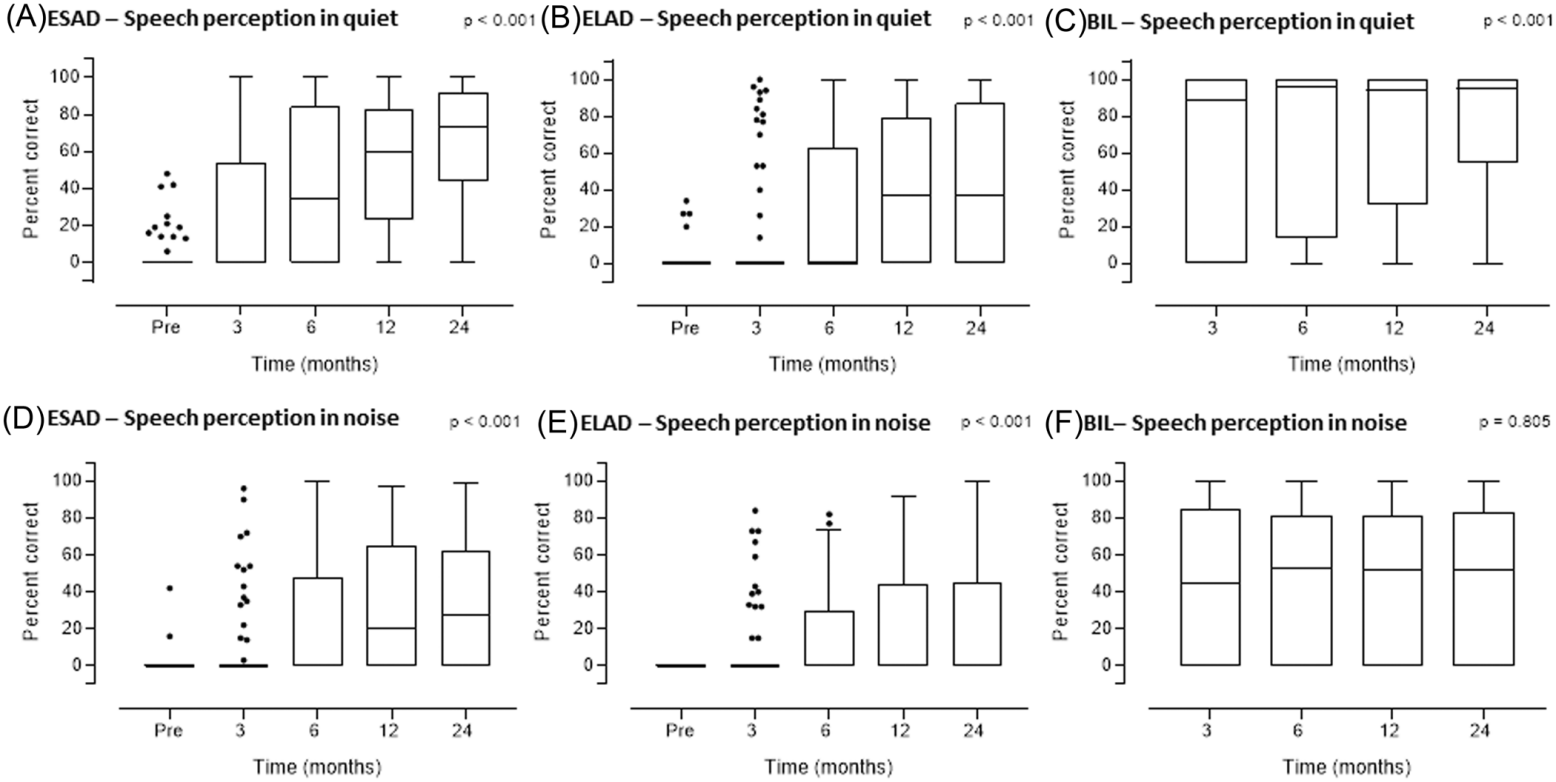
Longitudinal speech recognition by condition. Sentence recognition in quiet (A‐C) and noise (D‐F) across 24 months for ESAD, ELAD, and the bilateral condition (BIL), showing significant improvement over time and bilateral superiority at 24 months. ELAD, ear with longer auditory deprivation; ESAD, ear with shorter auditory deprivation.

Longitudinal analysis (Figure 4) revealed progressive improvement in sentence recognition for both ESAD and ELAD over the 24‐month period. In quiet, the median scores at 24 months were 73% for ESAD (IQR 44‐92), 37% for ELAD (IQR 0‐87), and 95% for BIL (IQR 55‐100) (*P* < .001).

In noise, the median scores were 27% for ESAD (IQR 0‐62), 0% for ELAD (IQR 0‐45), and 52% for BIL (IQR 0‐83). Notably, there was no significant improvement in BIL performance in noise between 3 and 24 months (*P* = .805). For both ears, performance stabilized after 12 months.

### Subgroups ≤10 Versus >10 Years Auditory Deprivation

**Figure 5:**
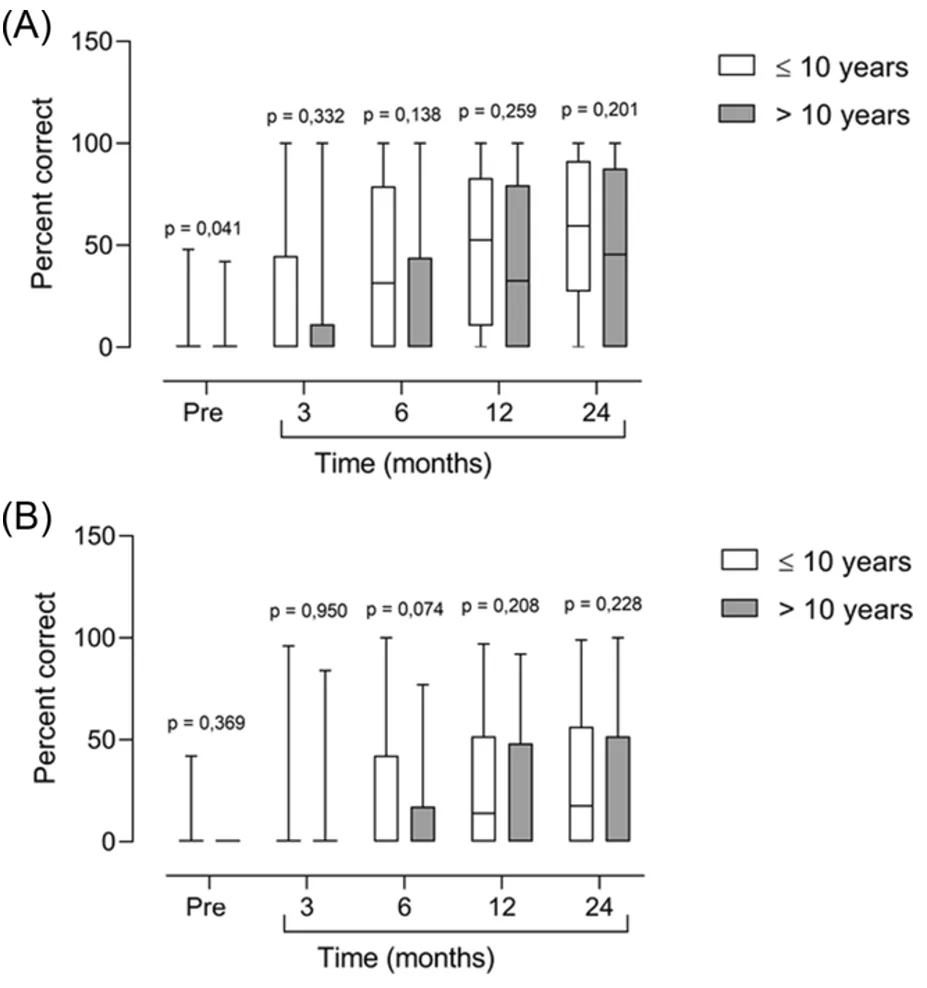
Ear‐level longitudinal sentence recognition stratified by deprivation duration (≤10 vs >10 years). (A) Quiet and (B) noise conditions over 24 months. No sustained longitudinal differences.

An ear‐level subgroup analysis (n = 126) stratified by deprivation duration (≤10 vs >10 years) was performed (Figure 5). No sustained longitudinal differences were identified in sentence recognition in quiet or noise over the 24‐month follow‐up period (all *P* > .05). Performance trajectories converged over time in both deprivation subgroups.

### Comparisons Between Time Points

No statistically significant differences were observed between the 12 and 24‐month time points for either condition (quiet or noise). For the BIL condition, a significant improvement in sentence recognition in quiet was noted between 3 and 24 months (*P* = .018), as shown in Tables 2 and 3.

**Table 2:** Sentence Recognition in Quiet Across Time Points: Median Values (IQR) and Post Hoc Pairwise Comparisons Using Dunn's Test

Descriptive statistics				Post hoc pairwise comparisons			
Time point	ESAD median (IQR)	ELAD median (IQR)	BIL median (IQR)	Comparisons	*P*‐value		
					ESAD	ELAD	BIL
Pre	0 (0‐0)	0 (0‐0)	‐	Pre vs 3 months	0.425	>0.999	‐
3 months	0 (0‐54)	0 (0‐0)	89 (0‐100)	Pre vs 6 months	<0.001	0.0043	‐
6 months	34 (0‐84)	0 (0‐63)	96 (14‐100)	Pre vs 12 months	<0.001	<0.001	‐
12 months	60 (23‐83)	37 (0‐79)	94 (32‐100)	Pre vs 24 months	<0.001	<0.001	‐
24 months	73 (44‐92)	37 (0‐87)	95 (55‐100)	3 months vs 6 months	0.058	0.143	>0.999
				3 months vs 12 months	<0.001	<0.001	>0.999
				3 months vs 24 months	<0.001	<0.001	0.018
				6 months vs 12 months	>0.999	0.242	>0.999
				6 months vs 24 months	<0.001	0.026	0.352
				12 months vs 24 months	0.166	>0.999	0.184

Abbreviations: BIL, bilateral condition; ELAD, ear with longer auditory deprivation; ESAD, ear with shorter auditory deprivation; IQR, interquartile range.

**Table 3:** Sentence Recognition in Noise Across Time Points: Median Values (IQR) and Post Hoc Pairwise Comparisons Using Dunn's Test

Descriptive statistics				Post hoc pairwise comparisons			
Time point	ESAD median (IQR)	ELAD median (IQR)	BIL median (IQR)	Comparisons	*P*‐value		
					ESAD	ELAD	BIL
Pre	0 (0‐0)	0 (0‐0)	‐	Pre vs 3 months	>0.999	>0.999	‐
3 months	0 (0‐0)	0 (0‐0)	45 (0‐84.5)	Pre vs 6 months	0.007	0.209	‐
6 months	0 (0‐48)	0 (0‐30)	53 (0‐81.5)	Pre vs 12 months	<0.001	0.001	‐
12 months	20 (0‐65)	0 (0‐44)	52 (0‐81.5)	Pre vs 24 months	<0.001	<0.001	‐
24 months	27 (0‐62)	0 (0‐45)	52 (0‐83)	3 months vs 6 months	0.260	>0.999	‐
				3 months vs 12 months	<0.001	0.143	‐
				3 months vs 24 months	<0.001	0.044	‐
				6 months vs 12 months	0.630	>0.999	‐
				6 months vs 24 months	0.013	0.519	‐
				12 months vs 24 months	>0.999	>0.999	‐

Abbreviations: BIL, bilateral condition; ELAD, ear with longer auditory deprivation; ESAD, ear with shorter auditory deprivation; IQR, interquartile range.

### Pure‐Tone Means and Speech Detection Thresholds

**Figure 6:**
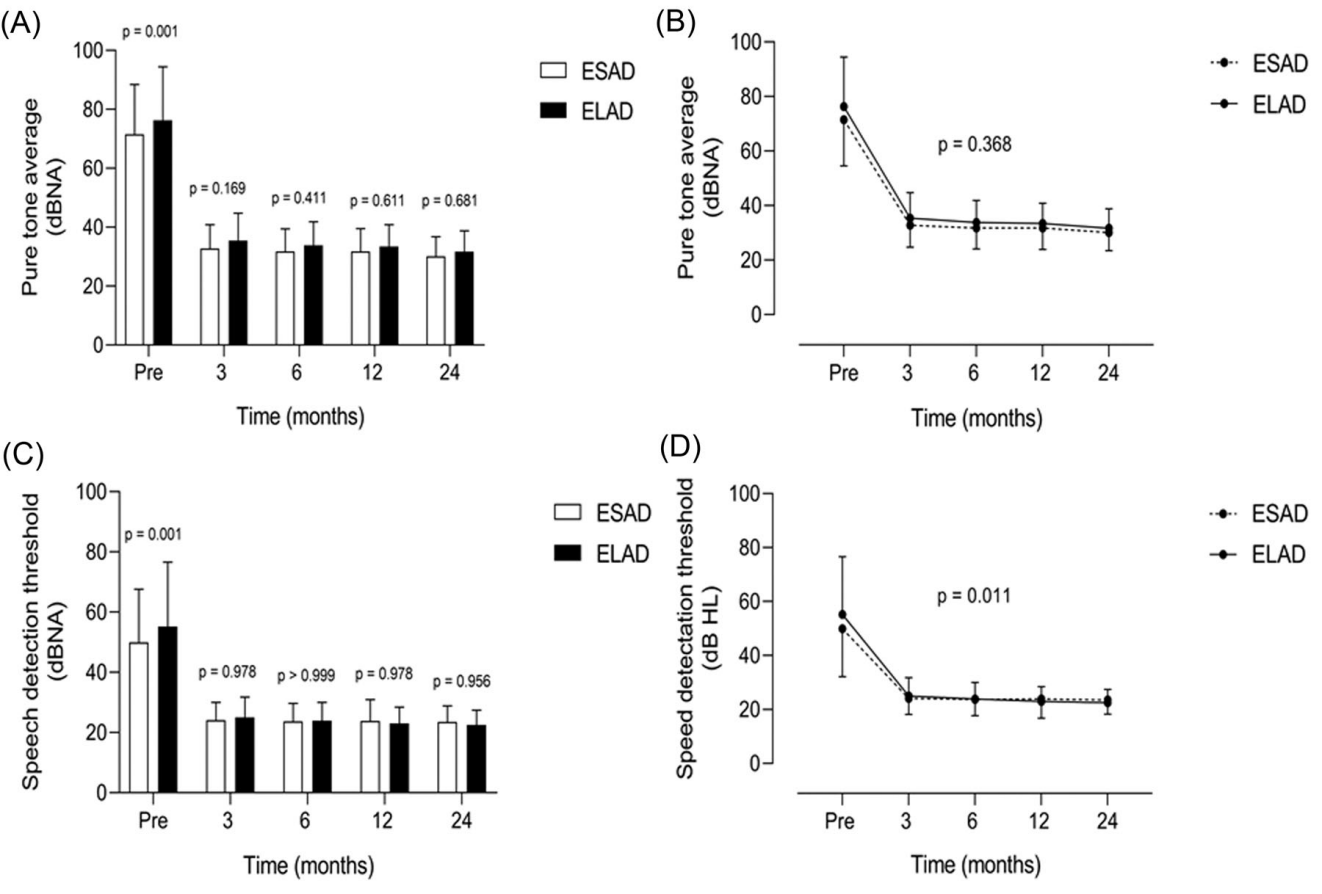
Longitudinal free‐field outcomes. (A, C) Main effect of ear (ESAD vs ELAD) for PTA and SDT. (B, D) Ear versus time interaction, illustrating differences in temporal trajectories between ears. ELAD, ear with longer auditory deprivation; ESAD, ear with shorter auditory deprivation; PTA, pure‐tone averages; SDT, speech detection thresholds.

Free‐field hearing thresholds and SDTs showed significant improvement beginning at 3 months postactivation, with stability maintained through 24 months (Figure 6).

In ESAD, the PTA improved from 71.5 to 30.1 dB HL, and the SDT from 49.9 to 23.6 dB HL. In ELAD, the PTA improved from 76.3 to 31.8 dB HL, and the SDT from 55.2 to 22.5 dB HL (*P* < .001 for all comparisons).

Two‐way repeated‐measures ANOVA demonstrated a significant main effect of ear (ESAD vs ELAD) for both PTA (Figure 6A) and SDT (Figure 6C). No significant interaction between ear and time was observed for PTA (Figure 6B), indicating parallel longitudinal trajectories. In contrast, SDT showed a significant interaction effect over time between ears (Figure 6D), reflecting different rates of improvement.

### Correlation Analysis

In the ESAD condition, sentence recognition in quiet was negatively correlated with the duration of prior hearing aid use (*r* = −0.293; *P* = .020) and positively correlated with the inter‐implant interval, both in quiet (*r* = 0.264; *P* = .037) and in noise (*r* = 0.285; *P* = .025). No significant correlations were found between the evaluated variables and auditory performance in the ELAD condition.

In the BIL condition, sentence recognition scores were negatively correlated with hearing aid use duration on the ESAD side (quiet: *r* = −0.312, *P* = .018; noise: *r* = −0.424, *P* = .001) and positively correlated with the inter‐implant interval (quiet: *r* = 0.450, *P* < .001; noise: *r* = 0.384, *P* = .003). Partial electrode insertion in the ELAD ear was also associated with poorer sentence recognition in quiet (*P* = .045) (Supplemental Tables S1‐6, available online).

## Discussion

This study demonstrated that, in a population of postlingually deafened undergoing sequential bilateral cochlear implantation, ESAD and ELAD exhibited significant auditory improvement over a 24‐month period. Free‐field hearing thresholds and speech detection thresholds improved significantly by 3 months postactivation and remained stable through 24 months. In both quiet and noise conditions, ESAD outperformed ELAD, consistent with previous findings.^3^, ^7^ Bilateral performance exceeded that of either monaural condition, regardless of the duration of auditory experience with ESAD. Notably, the inter‐implant interval showed a positive correlation with bilateral sentence recognition scores in both quiet and noise, suggesting that extended auditory experience with ESAD may partially compensate for the limited performance of ELAD.

Although no correlation was observed between the duration of sound deprivation and sentence recognition scores in quiet or noise over the 24‐month period, similar to findings showing no correlation between deprivation time and speech recognition within 3 years,^4^ the fact that ESAD presented better results than ELAD corroborates the literature, which shows that the duration of deafness is a negative predictor of speech perception.^2^ Nevertheless, this effect appears to be attenuated by the duration of auditory experience following implantation, as evidenced here by the positive correlation between inter‐implant interval and sentence recognition.^2^, ^8^

The absence of sustained longitudinal differences when stratifying ears by a 10‐year deprivation cutoff suggests that deprivation effects may operate along a continuum rather than through discrete temporal thresholds. Bernhard et al reported a graded association between deprivation duration and performance, supporting a continuous rather than dichotomous effect.^2^ Additionally, Sorrentino et al observed that even patients with deprivation exceeding 15 years may achieve meaningful postoperative gains, particularly when adequate auditory experience follows implantation.^9^

The results confirm that even ears with long periods of auditory deprivation and extended inter‐implant intervals can derive significant bilateral benefit, particularly in speech recognition in quiet. These findings suggest that long intervals of sound deprivation do not impede the benefit of bilaterality, but may be delayed due to the limitation of the ELAD adaptation speed, probably secondary to reduced neural plasticity after long periods of sound deprivation.^10^, ^11^

Although bilateral listening outperformed monaural conditions, speech perception in noise improved early and then stabilized. From a neurophysiological perspective, this plateau may reflect constraints in binaural encoding under persistent interaural asymmetry. Binaural integration depends on balanced excitation–inhibition mechanisms within the lateral superior olive, where interaural level differences and envelope timing cues are encoded.^12^ Asymmetric auditory deprivation can disrupt temporal precision, neural synchrony, and dynamic range balance between ears, reducing the fidelity of binaural cue representation. Experimental and modeling data suggest that such interaural mismatch degrades binaural summation and squelch mechanisms, particularly in complex acoustic environments.^12^ In sequential implantation, persistent ESAD‐ELAD asymmetry may therefore impose a physiological ceiling on binaural processing in noise, leading to early stabilization of BIL performance despite continued device use.

In comparison to previous longitudinal studies in postlingually deafened,^9^ which demonstrated auditory benefits even in patients with more than 15 years of bilateral deprivation, our findings further support that duration of deprivation should not be considered an isolated contraindication to cochlear implantation. However, in the context of sequential implantation, asymmetry in deprivation time poses a challenge to bilateral integration, which appears to require not only time but also extensive auditory experience with the first implanted ear.

No correlation was found between sentence recognition and either age or meningitis etiology. Our results are consistent with previous studies that found no significant effect of age after adjusting for deprivation time and CI experience.^4^, ^13^ Regarding meningitis, the lack of correlation in our cohort may be attributed to variability in the degree of cochlear ossification, which was not specifically stratified in this study. Hearing outcomes in adults postmeningitis depend on ossification extent, duration of deafness, electrode insertion depth, and neurological sequelae.^14^

Interestingly, we observed a negative correlation between the duration of hearing aid use and sentence recognition performance for both ESAD and BIL conditions. This contrasts with previous studies suggesting either positive or neutral effects of prior hearing aid use.^7^, ^15^ One plausible explanation is that patients in our cohort continued using hearing aids even in the absence of significant benefit, delaying timely bilateral electrical stimulation. This highlights the importance of evaluating the functional benefit of hearing aid use, rather than its duration alone, to determine the optimal timing for cochlear implantation.

Partial electrode insertion in ELAD was associated with poorer performance in sentence recognition in quiet under the BIL condition, reinforcing the need for full cochlear coverage to achieve optimal bilateral outcomes, especially in ears with long‐term deprivation.^16^, ^17^ Nevertheless, bilateral performance remained superior overall, suggesting that auditory experience with ESAD may play a more critical compensatory role than electrode insertion depth in ELAD.

Among the limitations of this study are its retrospective design, which is subject to potential selection bias and sample heterogeneity. Manufacturer‐specific subgroup analyses were not performed, given the primary focus on deprivation‐related variables and potential confounding by device heterogeneity over time. Additionally, CI electrode designs, processor technologies, and rehabilitation protocols evolved over the study period, and era‐ or technology‐specific subgroup analyses were not feasible due to small and uneven subgroup sizes. The definition of auditory deprivation was based on the duration of severe‐to‐profound hearing loss and documented hearing aid use, which may not fully capture qualitative inter‐individual differences in functional aided benefit. In addition, bilateral auditory outcomes were assessed using sentence recognition in quiet and noise, as spatial hearing and sound localization measures were not systematically available in this retrospective cohort. Therefore, the present findings primarily reflect bilateral speech perception outcomes rather than spatial auditory performance. Future prospective multicenter studies across different linguistic and cultural settings are warranted to further explore the influence of deprivation duration, aided benefit, spatial hearing, and to confirm the generalizability of the present findings.

## Conclusion

This longitudinal study demonstrated that the implanted ear with the shortest duration of auditory deprivation exhibited superior speech recognition performance, particularly in quiet environments, compared to the ear with the longest deprivation. Nonetheless, simultaneous use of 2 CIs resulted in better auditory outcomes than either ear alone, even in cases with substantial asymmetry in deprivation duration.

Additionally, prior hearing aid use without meaningful functional benefit was associated with poorer auditory outcomes, suggesting that prolonged reliance on ineffective acoustic amplification may delay optimal timing for bilateral electrical stimulation. Partial electrode insertion in the more deprived ear negatively impacted bilateral speech recognition, underscoring the importance of achieving full cochlear coverage whenever anatomically feasible. In contrast, greater auditory experience with the first implant emerged as a positive contributor to overall performance.

These findings indicate that prolonged auditory deprivation should not be considered a contraindication to second‐side implantation. However, minimizing interaural asymmetry through timely second implantation, ensuring full electrode insertion, and critically evaluating aided functional benefit are essential factors to optimize bilateral outcomes in sequential cochlear implantation.

## Author Contributions

**Eduardo S. Farinazzo**, conceptualization, methodology, data curation, formal analysis, writing (original draft), writing (review and editing). **Rubens V. de Brito Neto**, formal analysis, writing (review and editing). **Elisabete H. Yamaguti**, data curation, formal analysis. **Marco A. Fornazieri**, formal analysis, writing (review and editing). **Luiz F. M. Lourençone**, conceptualization, methodology, supervision, validation, writing (review and editing).

## Disclosures

### Competing interests

None.

### Funding source

None.

## Supporting information


**Supplementary Table 1. Correlation analysis in ESAD.** Spearman correlations between sentence recognition (quiet and noise) and age, auditory deprivation duration, prior hearing aid use, and inter‐implant interval.Abbreviation: ESAD= ear with shorter auditory deprivation
**Supplementary Table 2. ESAD performance by subgroups.** Mann–Whitney comparisons of sentence recognition (quiet and noise) according to sex, etiology, and electrode insertion.Abbreviation: ESAD= ear with shorter auditory deprivation
**Supplementary Table 3. Correlation analysis in ELAD.** Spearman correlations between sentence recognition (quiet and noise) and age, auditory deprivation duration, prior hearing aid use, and inter‐implant interval.Abbreviation: ELAD = ear with longer auditory deprivation
**Supplementary Table 4. ELAD performance by subgroups.** Mann–Whitney comparisons of sentence recognition (quiet and noise) according to sex, etiology, and electrode insertion.Abbreviation: ELAD = ear with longer auditory deprivation
**Supplementary Table 5. Correlation analysis in BIL.** Spearman correlations between sentence recognition (quiet and noise) and age, auditory deprivation, prior hearing aid use (ESAD and ELAD), and inter‐implant interval.Abbreviations: BIL= bilateral condition; ESAD= ear with shorter auditory deprivation; ELAD = ear with longer auditory deprivation
**Supplementary Table 6. Subgroup comparisons in BIL.** Mann–Whitney analysis of sentence recognition (quiet and noise) according to sex, etiology, and electrode insertion in ESAD and ELAD.Abbreviations: BIL= bilateral condition; ESAD= ear with shorter auditory deprivation; ELAD = ear with longer auditory deprivation.

## References

[1] Sladen DP , Zappler A . Older and younger adult cochlear implant users: speech recognition in quiet and noise, quality of life, and music perception. Am J Audiol. 2015;24(1):31‐39. 10.1044/2014_AJA-13-0066 25239296

[2] Bernhard N , Gauger U , Romo Ventura E , et al. Duration of deafness impacts auditory performance after cochlear implantation: a meta‐analysis. Laryngoscope Investig Otolaryngol. 2021;6(2):291‐301. 10.1002/lio2.528 PMC8035957 33869761

[3] Boisvert I , McMahon CM , Dowell RC . Long‐term monaural auditory deprivation and bilateral cochlear implants. Neuroreport. 2012;23(3):195‐199. 10.1097/WNR.0b013e32834fab4b 22182978

[4] Medina MM , Polo R , Gutierrez A , et al. Cochlear implantation in postlingual adult patients with long‐term auditory deprivation. Otol Neurotol. 2017;38(8):e248‐e252. 10.1097/MAO.0000000000001257 28806334

[5] Brazilian Ministry of Health. Ordinance No. 2, Consolidation Ordinance No. 2/GM/MS (September 28, 2017), incorporating the cochlear implant candidacy criteria established in Ordinance No. 2,776 (2014). Brasília, Brazil.

[6] Oliveira ST. Avaliação da percepção de fala utilizando sentenças do dia a dia [dissertation]. São Paulo, Brazil: Pontifícia Universidade Católica de São Paulo; 1992.

[7] Smulders YE , Hendriks T , Stegeman I , et al. Predicting sequential bilateral cochlear implantation performance in postlingually deafened adults: a retrospective cohort study. Clin Otolaryngol. 2018;43(6):1500‐1507. 10.1111/coa.13193 30022607

[8] Heutink F , Verbist BM , van der Woude WJ , et al. Factors influencing speech perception in adults with a cochlear implant. Ear Hear. 2021;42(4):949‐960. 10.1097/AUD.0000000000000988 33480623 PMC8221708

[9] Sorrentino F , Gheller F , Lunardi G , et al. Cochlear implantation in adults with auditory deprivation: what do we know about it? Am J Otolaryngol. 2020;41(2):102366. 10.1016/j.amjoto.2019.102366 31837837

[10] Sun Y , Dillier N , Yeagle J , et al. Cortical plasticity in post‐lingually deafened adults with cochlear implants: a longitudinal fMRI study. Neuroimage Clin. 2021;30:102640. 10.1016/j.nicl.2021.102640 33799272 PMC8044711

[11] Fallon JB , Shepherd RK , McKay CM . Contribution of auditory brain plasticity to cochlear implant benefits: a review of animal studies. Trends Neurosci. 2008;31(3):111‐118. 10.1016/j.tins.2008.01.004

[12] Anderson SR , Burg E , Suveg L , Litovsky RY . Review of binaural processing with asymmetrical hearing outcomes in patients with bilateral cochlear implants. Trends Hear. 2024;28:23312165241229880. 10.1177/23312165241229880 38545645 PMC10976506

[13] Reeder RM , Firszt JB , Holden LK , Strube MJ . A longitudinal study in adults with sequential bilateral cochlear implants: time course for individual ear and bilateral performance. J Speech Lang Hear Res. 2014;57(3):1108‐1126. 10.1044/2014_JSLHR-H-13-0087 24686778 PMC4057980

[14] Alhabib S , Alzhrani F , Albadr F , et al. Cochlear implantation in bacterial meningitis: a case series. J Laryngol Otol. 2021;135(9):797‐803. 10.1017/S0022215121001601

[15] Park HK , Lee JH , Lee KY , et al. Long‐term benefits of hearing aid use prior to cochlear implantation on speech perception. Sci Rep. 2018;8(1):1226. 10.1038/s41598-018-19613-w 29352239

[16] Finley CC , Holden TA , Holden LK , et al. Role of electrode placement as a contributor to variability in cochlear implant outcomes. Otol Neurotol. 2008;29(7):920‐928. 10.1097/MAO.0b013e318184f492 18667935 PMC2663852

[17] O'Connell BP , Cakir A , Hunter JB , et al. Electrode location and angular insertion depth are predictors of audiologic outcomes in cochlear implantation. Otol Neurotol. 2016;37(8):1016‐1023. 10.1097/MAO.0000000000001125 27348391 PMC4983244

