# Supplementary material for: Effects of Asymmetric Auditory Deprivation in Sequential Cochlear Implant
Source: Otolaryngol Head Neck Surg. 2026 Jun 3;175(3):672–81. doi: 10.1002/ohn.70308 (PMC13525876; doi:10.1002/ohn.70308)
Supplement: Supplementary file 1 [file OHN-175-672-s001.docx]

| **Supplementary Table 1** |  |  |  |
| --- | --- | --- | --- |
| **Variables** | **r** | **p** |  |
| Sentences in quiet ESAD  x Age | -0.081 | 0.526 |  |
| Sentences in quiet ESAD  x  Auditory deprivation (ESAD) | -0.142 | 0.268 |  |
| Sentences in quiet ESAD  x Hearing age (ESAD) | -0.293 | 0.020 |  |
| Sentences in quiet ESAD  x Interimplants time | 0.264 | 0.037 |  |
| Sentences in noise ESAD  x Age | -0.165 | 0.196 |  |
| Sentences in noise ESAD  x  Auditory deprivation (ESAD) | 0.076 | 0.553 |  |
| Sentences in noise ESAD  x Hearing age (ESAD) | -0.215 | 0.091 |  |
| Sentences in noise ESAD  x Interimplants time | 0.285 | 0.025 |  |

| **Supplementary Table 2** | | | | | |
| --- | --- | --- | --- | --- | --- |
| **Sentences in quiet ESAD** | Sex |  | **Male** | **Female** | **p** |
|  |  | IQ 25% | 39 | 48.5 | 0.922 |
|  |  | Median | 73 | 74 |  |
|  |  | IQ 75% | 91 | 92 |  |
|  | Etiology |  | **Meningitis** | **Others** | **p** |
|  |  | IQ 25% | 0 | 50 | 0.251 |
|  |  | Median | 0 | 75 |  |
|  |  | IQ 75% | 73 | 92 |  |
|  | Electrode Insertion |  | **Partial** | **Total** | **p** |
|  |  | IQ 25% | 43.5 | 41.5 | 0.864 |
|  |  | Median | 75 | 73 |  |
|  |  | IQ 75% | 85 | 92.5 |  |
| **Sentences in noise ESAD** | Sex |  | **Male** | **Female** | **p** |
|  |  | IQ 25% | 0 | 0 | 0.961 |
|  |  | Median | 27 | 29 |  |
|  |  | IQ 75% | 68 | 61.5 |  |
|  | Etiology |  | **Meningitis** | **Others** | **p** |
|  |  | IQ 25% | 0 | 0 | 0.516 |
|  |  | Median | 10 | 31 |  |
|  |  | IQ 75% | 67.5 | 60 |  |
|  | Electrode Insertion |  | **Partial** | **Total** | **p** |
|  |  | IQ 25% | 0 | 0 | 0.499 |
|  |  | Median | 9.5 | 29 |  |
|  |  | IQ 75% | 50.75 | 64 |  |

| **Supplementary Table 3** |  |  |  |
| --- | --- | --- | --- |
| **Variables** | **r** | **p** |  |
| Sentences in quiet ELAD x Age | 0.158 | 0.217 |  |
| Sentences in quiet ELAD x  Auditory deprivation (ELAD) | -0.065 | 0.611 |  |
| Sentences in quiet ELAD x Hearing aids (ELAD) | 0.106 | 0.407 |  |
| Sentences in quiet ELAD x Interimplants time | 0.158 | 0.217 |  |
| Sentences in noiseELAD x Age | 0.168 | 0.188 |  |
| Sentences in noiseELAD x  Auditory deprivation (ELAD) | -0.013 | 0.922 |  |
| Sentences in noiseELAD x Hearing aids (ELAD) | 0.052 | 0.688 |  |
| Sentences in noiseELAD x Interimplants time | 0.144 | 0.260 |  |

| **Supplementary Table 4** | | | | | |
| --- | --- | --- | --- | --- | --- |
| **Sentences in quiet ELAD** | Sex |  | **Male** | **Female** | **p** |
|  |  | IQ 25% | 0 | 0 | 0.581 |
|  |  | Median | 31 | 45 |  |
|  |  | IQ 75% | 75 | 91.5 |  |
|  | Etiology |  | **Meningitis** | **Others** | **p** |
|  |  | IQ 25% | 0 | 0 | 0.151 |
|  |  | Median | 0 | 49 |  |
|  |  | IQ 75% | 73 | 88 |  |
|  | Eletrodes insertation |  | **Partial** | **Total** | **p** |
|  |  | IQ 25% | 0 | 0 | 0.123 |
|  |  | Median | 0 | 45 |  |
|  |  | IQ 75% | 26 | 87.75 |  |
| **Sentences in noise ELAD** | Sex |  | **Male** | **Female** | **p** |
|  |  | IQ 25% | 0 | 0 | 0.630 |
|  |  | Median | 0 | 0 |  |
|  |  | IQ 75% | 43 | 53.25 |  |
|  | Etiology |  | **Meningitis** | **Others** | **p** |
|  |  | IQ 25% | 0 | 0 | 0.480 |
|  |  | Median | 0 | 0 |  |
|  |  | IQ 75% | 29 | 46 |  |
|  | Eletrodes insertation |  | **Partial** | **Total** | **p** |
|  |  | IQ 25% | 0 | 0 | 0.266 |
|  |  | Median | 0 | 0 |  |
|  |  | IQ 75% | 0 | 45.75 |  |

| **Supplementary Table 5** |  |  |  |
| --- | --- | --- | --- |
| **Variables** | **r** | **p** |  |
| Sentences in quiet BIL x Age (ESAD) | -0.250 | 0.061 |  |
| Sentences in quiet BIL x Age (ELAD) | -0.078 | 0.564 |  |
| Sentences in quiet BIL x  Auditory deprivation (ESAD) | 0.142 | 0.293 |  |
| Sentences in quiet BIL x  Auditory deprivation (ELAD) | 0.08 | 0.556 |  |
| Sentences in quiet BIL x Hearing aids (ESAD) | -0.312 | 0.018 |  |
| Sentences in quiet BIL x Hearing aids (ELAD) | -0.152 | 0.258 |  |
| Sentences in quiet BIL x Interimplants time | 0.450 | < 0.001 |  |
| Sentences in noise BIL x Age (ESAD) | -0.244 | 0.067 |  |
| Sentences in noise BIL x Age (ELAD) | -0.086 | 0.525 |  |
| Sentences in noise BIL x  Auditory deprivation (ESAD) | 0.032 | 0.812 |  |
| Sentences in noise BIL x  Auditory deprivation (ELAD) | 0.045 | 0.739 |  |
| Sentences in noise BIL x Hearing aids (ESAD) | -0.424 | 0.001 |  |
| Sentences in noise BIL x Hearing aids (ELAD) | -0.234 | 0.080 |  |
| Sentences in noise BIL x Interimplants time | 0.384 | 0.003 |  |

| **Supplementary Table 6** | | | | | | |
| --- | --- | --- | --- | --- | --- | --- |
| **Sentences in quiet BIL** | Sex |  | **Male** | **Female** | **p** | |
|  |  | IQ 25% | 56.5 | 80.25 | 0.993 | |
|  |  | Median | 98 | 95 |  |  |
|  |  | IQ 75% | 100 | 100 |  |  |
|  | Etiology |  | **Meningitis** | **Others** | **p** | |
|  |  | IQ 25% | 11.5 | 77.25 | 0.900 | |
|  |  | Median | 100 | 96 |  |  |
|  |  | IQ 75% | 100 | 100 |  |  |
|  | Electrode Insertion ESAD |  | **Partial** | **Total** | **p** | |
|  |  | IQ 25% | 65.5 | 76 | 0.970 | |
|  |  | Median | 90.5 | 97 |  |  |
|  |  | IQ 75% | 100 | 100 |  |  |
|  | Electrode Insertion ELAD |  | **Partial** | **Total** | **p** | |
|  |  | IQ 25% | 0 | 80 | 0.045 | |
|  |  | Median | 30.5 | 98 |  |  |
|  |  | IQ 75% | 61 | 100 |  |  |
| **Sentences in noise BIL** | Sex |  | **Male** | **Female** |  | **p** |
|  |  | IQ 25% | 10 | 31.75 |  | 0.365 |
|  |  | Median | 55 | 65.5 |  |  |
|  |  | IQ 75% | 78.5 | 90.75 |  |  |
|  | Etiology |  | **Meningitis** | **Others** |  | **p** |
|  |  | IQ 25% | 0 | 31.75 |  | 0.481 |
|  |  | Median | 47 | 63 |  |  |
|  |  | IQ 75% | 93.5 | 83.75 |  |  |
|  | Electrode Insertion ESAD |  | **Partial** | **Total** |  | **p** |
|  |  | IQ 25% | 0 | 26 |  | 0.118 |
|  |  | Median | 44.5 | 65 |  |  |
|  |  | IQ 75% | 57.5 | 90 |  |  |
|  | Electrode Insertion ELAD |  | **Partial** | **Total** |  | **p** |
|  |  | IQ 25% | 0 | 30 |  | 0.053 |
|  |  | Median | 0 | 61 |  |  |
|  |  | IQ 75% | 0 | 89 |  |  |
